# Acknowledgement to Reviewers of *Materials* in 2013

**DOI:** 10.3390/ma7031444

**Published:** 2014-02-25

The editors of *Materials* would like to express their sincere gratitude to the following reviewers for assessing manuscripts in 2013:

**Table:** 

Abdallh, Ahmed Mohamed A.	Alessandri, Ivano	Arthur, Timothy S.
Abdul-Aziz, Ali	Allam, Nageh K.	Asano, Kohta
Abichou, Tarek	Almond, Darryl P.	Askes, Harm
Abidi, Noureddine	Alonso-Lomillo, M. Asunción	Asmussen, Jes
Abot, Jandro L.	Altintas, Zeynep	Asthana, Rajiv
Acet, M.	Ambrosi, Davide	Atkinson, John David
Achilias, Dimitris S.	Aminorroaya, Sima	Atrei, Andrea
Adamopoulos, George	Anagiotos, Andreas	Atsushi, Iizuka
Adele, Carrado	Andersen, Otto	Aydil, Eray S.
Afazov, S.M.	Andersen, Svend Olav	Ayllon, Jose-Antonio
Afzal, Muhammad	Andraud, Chantal	Bachmann, Julien
Aggelis, Dimitrios G.	Aneziris, Christos G.	Bacsa, Revathi R.
Agnelli, Silvia	Angove, Michael	Bader, Rainer
Ago, Mariko	Anifantis, N.K.	Bae, Youn-Sang
Ahmari, Saeed	Anker, Jeffrey N.	Baglioni, Piero
Aigner, Achim	Ansari, Imtiyaz Ahmed	Bailey, Stuart
Aikawa, Shinya	Ansell, Martin P.	Bakar, Mohamed
Ajami, Elnaz	Antolovich, Stephen D.	Balcom, Bruce
Ajersch, Frank	Anzai, Jun-ichi	Balzani, Daniel
Akbar, Sheikh A.	Aoyagi, Satoka	Banerjee, Raj
Al-Obaidi, Hisham	Ardelean, Ion	Bank, Craig E.
Al-Tabbaa, Abir	Arencibia, Amaya	Bao, Ningzhong
Alam, M. Shahria	Argyropoulos, Dimitris S.	Bao, Xiulong
Alavarez, Laurent	Ariga, Katsuhiko	Barbero, Basilio Ramos
Albonetti, Stefania	Arjona-Lopez, Marco Antonio	Barkey, Mark
Albrecht, Markus	Arnold, Cris	Barnard, Amanda S.
Barsoum, Michel	Bohner, Marc	Cao, Zhiqiang
Bartali, R.	Bojdys, Michael J.	Carbone, Giuseppe
Barter, Simon	Boller, Herbert	Carbonell, Jorge
Bartolome, Juan	Borisov, S.M.	Cascales, Concepción
Bastos, Alexandre	Borrmann, Thomas	Caslake, Laurie
Bates, David	Bos, Jan-Willem	Casson, Alexander
Batis, George	Bottino, Marco C.	Castro Martin, Maria Yolanda
Battezzati, Livio	Boud, Fathi	Castro-Gomes, Joao
Bäuerle, Peter	Boussuge, Michel	Castro, Ricardo
Bax, Daniel V.	Boutejdar, Ahmed	Castrucci, Paola
Baxavanis, Theocharis	Boyter, Henry	Casu, M. Benedetta
Bazant, Martin Z.	Brailovski, V	Cavagnaro, Marta
Bazylinski, Dennis A.	Brambilla, Davide	Chadov, Stanislav
Beattie, James K.	Branco, Fernando	Chagnon, Gregory
Beckermann, Christoph	Brandt, Milan	Chalivendra, Vijay
Behrens, Malte	Braun, Artur	Challamel, Noël
Belfort, Georges	Braun, Ulrike	Chan, Daniel
Belonoshko, Anatoly B.	Breadmore, Michael	Chang, Min Hsing
Beltramini, Jorge Norberto	Bretcanu, Oana	Chang, Yu-Chao
Belviso, Claudia	Bringas, Eugenio	Chauhan, Bhanu
Bendavid, Avi	Brischke, Christian	Chaurand, Pierre
Benedetti, M. Di	Brodkorb, Andre	Chen, Bo
Bensch, Wolfgang	Brossmann, Ulrich	Chen, Zhi David
Berglund, Lars	Brostow, Wiltold	Chen, I-Chen
Bergmann, Christian	Brousseau, Emmanuel	Chen, J.K.
Bergmann, M.E. Henry	Brovarone, C. Vitale	Chen, Jem-kun
Bernal López, Susan A.	Brun, Nicolas	Chen, Liang-yih
Berry, John	Bruns, Nico	Chen, Luwei
Bessière, Aurélie	Bryant, Gary	Chen, Min
Bethell, Donald	Buchwald, Anja	Chen, Shen-Ming
Bhattacharjee, Sudip	Bunning, Timothy J.	Chen, Tao
Bi, Lei	Burton, Zachary F.	Chen, Yongsheng
Biermann, Dirk	Cabrales, Luis	Chen, Zuliang
Bikiaris, Dimitrios	Calin, Mariana	Cheng, Ming-cheng
Bing, Kong Ling	Callum, Hill	Cheng, Yang-tse
Binnemans, Koen	Calnan, Sonya	Cheng, Zhengdong
Binner, Jon	Cam, Jean-Benoît Le	Cherif, Chokri
Biris, Alexandru S.	Cambier, Francis	Chew, Huck Beng
Birnie, Dunbar P.	Camblor, Miguel A.	Chi, Ki-whan
Blackburn, Jeffrey L.	Campbell, John	Chica, Antonio
Blanche, Pierre-alexandre	Cannillo, Valeria	Chiodo, Letizia
Blau, Werner	Cao, Peng	Chmielarz, Lucjan
Chokkalingham, Anand	Dalgarno, Kenneth	Dorozhkin, Sergey V.
Chowdhury, Sanjib	Dammaschke, Till	Douki, Thierry
Christensen, Bjørn Erik	Das, Amit	Douroumis, Dennis
Chung, Kwok L.	Das, Hena	Dovichi, Norman J.
Chung, Wenchiang R.	Das, Suman	Driver, Julian
Church, Tamara L.	Datashvili, Tea	Duance, Victor
Churchill, Hugh	Datye, Abhaya	Dubowski, Jan J.
Chwee Sim, Goh	David, Allan E.	Dudziak, Tomasz
Ciardelli, Francesco	Davidovits, Joseph	Dufès, Christine
Ciarletta, Pasquale	Davidson, Mark	Dujardin, Christophe
Cincotti, Alberto	Davidson, Paul	Dunbar, Alan
Cirillo, Giuseppe	Davim, Paulo	Durual, Stéphane
Ciurana, Joaquim	Davis, W. Rhett	Dziubla, Thomas
Clarke, Anthony	Davit, Yohan	Ebel, Thomas
Claus, Claus	De Aza Moya, Piedad	Ebong, Abasifreke
Clegg, Dennis	De Belie, Nele	Edrisy, Afsaneh
Clench, Malcolm	De Buysser, Klaartje	Ehrich, Marion
Cleo, Choong	De Brito, Jorge	Eichen, Yoav
CLEVER, Guido	De Cos, Maria Elena	El-Kaderi, Hani M.
Cochran, Eric W.	De la Chapelle, Marc Lamy	El-Shafei, Ahmed
Cojocaru-Mirédin, Oana	De Lima, Renata Cristina	Elkedim, O.
Colangelo, Francesco	De Rooij, Mario	Enfedaque, Alejandro
Collins, Frank	De Tommasi, Domenico	Englander, Ongi
Collins, Frank G.	De Vos, Paul	Englund, Karl R.
Conde, J.P.	Debliquy, Marc	Ennas, Guido
Cook, Wayne	Decoste, Jared B	Entrop, A.G.
Cooper, Paul	Deflorian, Flavio	Er, Süleyman
Corma, Avelino	Defouw, John	Erathodiyil, Nandanan
Corriou, Jean-Pierre	DeGroot, Marty W.	Erdem, Emre
Costa, Filippo	Del Hierro, Isabel	Escobar, Gustavo
Cousins, Brian G.	Delacourt, Charles	Espallargas, S.J. García
Couteau, Céline	Delai Sun, Darren	Etches, Julie
Creus, Juan	Della Ciana, Leopoldo	Etienne, Mathieu
Criado, M.	Della Ventura, Giancarlo	Facchetti, Antonio
Crotti, Tania	Denicourt-Nowicki, Audrey	Fahrenholtz, William G.
Cuniberti, Gianaurelio	Dhakal, H.N.	Fahrmann, Michael
Czigany, T.	Dias, Salvador	Falcaro, Paolo
Czujko, T.	Díez-Pascual, Ana M.	Falciglia, Pietro P.
Da’na, Enshirah	Dimitrijevic, Nada M.	Fan, Mizi
Dagorne, Samuel	Ding, Zhifeng	Fang, Ning
Dai, Jian-guo	Dolado, Jorge S.	Fare, Silvia
Dai, Ying	Dong, Chensong	Farhat, Mohamed
Farid, Mohammed M.	Gherasoiu, Iulian	Guo, Zhanhu
Faruk, Omar	Ghorbani, Kamran	Guo, Ting
Felser, Claudia	Ghosh, Gargi	Guo, Youguang
Fernandes, Francisco	Ghrib, Faouzi	Gupta, Mool C.
Fernandez, Irene	Gibbons, Greg	Gupta, Rajeev
Fernandez, Pablo Lodeiro	Gibson, Mark	Gutiérrez, Humberto R.
Ferretti, Valeria	Ginosar, Dan	Haastert-Talini, Kirsten
Fierro, J.L.G.	Girard-Lauriault, Pierre-Luc	Hagelberg, Frank
Filonovich, Sergej	Girousi, Stella	Hagstroem, Bengt
Fischer, Holger	Gläser, Roger	Hahn, David W.
Fischetti, Massimo V.	Glowacki, Bartek A.	Haj-Ali, Rami
Follain, Nadège	Goddard, William A., III	Hall, Lisa
Fonseca, J.L.C.	Golañski, G.	Hallström, Stefan
Foroughi, Javad	Golden, Teresa Diane	Haluska, Miroslav
Foster, Michelle T.	Golombek, Klaudiusz	Hameed, Nishar
Fourie, Coenrad	González, S.	Hamilton, Carter
Frankcombe, Terry	Goodell, Barry	Han, Ang Wee
Frazier, Charles	Goodship, Vannessa	Han, Jung-Suk
Frédéric, Debeaufort	Gotoh, Kazuma	Hanemann, Thomas
Freeman, Harold	Goychuk, Igor A.	Hao, Yang
Frigione, Mariaenrica	Grady, Brian	Harbrecht, B.
Frontera, Patrizia	Graeve, Olivia A.	Harding, Ian
Froumin, N.	Granchi, Donatella	Harimkar, Sandip
Fuhrmann-Lieker, Thomas	Grant, David	Harris, Vincent
Fukada, Toshiyuki	Gray, Derek G.	Harrysson, Ola
Fukuda, Takamitsu	Greluk, Magdalena	Hartwig, Thomas
Fukui, Kunihiro	Grevey, Dominique	Harutyunyan, Avetik R.
Gabriele, Bartolo	Griesser, Hans	Hasché, Frédéric
Gaitzsch, Uwe	Griffiths, Christian	Hata, Kenji
Gales, Luís	Grimaldi, C.	Hauert, Roland
Gandolfi, Maria Giovanna	Grimsdale, Andrew Clive	Hayakawa, Tomokatsu
Gao, Yonggui	Grinberg, Marek	Heap, Michael J.
Gardelis, Spiros	Grujicic, M.	Hector, Andrew
Gardner, Diane	Gu, Frank	Heinemann, Sascha
Garrido, Mário	Gualtieri, Alessandro F.	Hempel, Ute
Garrone, Edoardo	Guay, Daniel	Henneberger, Fritz
Gashti, Mazeyar Parvinzadeh	Gubner, Rolf	Her, Shiuh-chuan
Gasparotto, Alberto	Guelcher, Scott A.	Herrera, José E.
Gassmann, Stefan	Guillon, Olivier	Hesemann, Peter
Gawande, Manoj B.	Gun’ko, Y. K.	Hezel, Dominik
Gentile, Gennaro	Gunasekaran, Alfred	Hickey, Owen
Gharghi, Majid	Gunnella, Roberto	Hickey, Stephen G.
Hickok, Noreen J.	Hutchison, Gary	Johnston, Carl Gordon
Hietschold, Michael	Hutmacher, Dietmar	Joly, Nicolas
Higashimoto, Shinya	Huynen, Isabelle	Jones, David
Higgins, Michael	Hwang, Jun-Dar	Jones, Jacob L.
Hills, Colin	Iafisco, Michele	Jones, Julian R.
Hinds, G.	Ibarra, M. Ricardo	Jonkers, Henk M.
Hippler, Rainer	Idapalapati, Sridhar	Jonoobi, Mehdi
Hiscox, Jennifer	Inal, Kaan	Jordan, Andrew N.
Ho, Ghim Wei	Infusino, M.	Jozwiak-Niedzwiedzka, Daria
Ho, Johnny Chung Yin	Ingham, Eileen	Jun, Beomcho
Ho, Wen-Fu	Iniesta, Jesús	Juodkazis, Saulius
Hodgson, Simon	Ionov, Leonid	Jurgens, Philipp
Hodzic, Alma	Irimia-Vladu, M.	Kadokawa, Jun-ichi
Hofmann, Michael	Ishibashi, Takayuki	Kaegi, Ralf
Högberg, Hans	Ishigaki, Tadashi	Kainer, Karl Ulrich
Hol, Myrthe K.S.	Iwamura, Munetaka	Kalantar-zadeh, Kourosh
Holley, Juliana Calabria	Iwasaki, Tomohiro	Kalaskar, Deepak
Holmström, Stefan	Iwasaki, Yasuhiko	Kaliappan, Muthukumar
Holten-Andersen, Niels	Izumi, Yasuo	Kamali-Bernard, Siham
Honda, Shinya	Jabbari, Mohammad Ali	Kamshilin, Alexei
Hong, Cheng-Shong	Jablonski, Paul D.	Kan, Daisuke
Hong, Liang	Jacquart, Sylvaine	Kandelbauer, Andreas
Hong, Yi	Jadwicienczak, Wojciech	Kang, Chulhun
Hossain, Khandaker M.A.	Jamet, Matthieu	Karouta, Fouad
Hou, Shifeng	Jarosz-Wilkolazka, Anna	Karpukhina, Natalia
Hou, Zhengmeng	Jayasinghe, Suwan	Kasi, Rajeswari M.
Houwman, Evert	Je, Jung Ho	Kaskel, Stefan
Hsiao, Kuang-Che	Jefferson, A.D.	Katsaras, John
Hsiao, Vincent K.S.	Jeng, Dong-Sheng	Katz, Jonathan I.
Hu, Jianjiang	Jeon, D.Y.	Kavanagh, John
Hu, Jinlian	Jeon, Han-Yong	Kawaguchi, Haruma
Huang, Chin-Pao	Jeong, Jung Hyun	Kawai, Tsuyoshi
Huang, Hai	Jeong, S.-J.	Kehoe, Sharon
Huang, Jie	Jha, Himendra	Keil, Frerich
Huang, Jun	Ji, Yaying	Kent, Michael
Huang, Wei Min	Jian, Sheng-rui	Kenyon, William J.
Hubbe, Martin A.	Jiang, Liudi	Kessel, David
Hughes, Taylor L.	Jiang, Xian	Khawa, T.S.
Hung, Wayne	Jo, Young Min	Khelifi, Samira
Hur, Janet I.	Jockusch, Steffen	Khlobystov, Andrei N.
Hurst, James K.	John, Kechagias	Khulbe, K.C.
Hutchinson, J.M.	Johnson, Steven	Kim, Byung-Sun
Kim, Changsoo	Kruse, Peter	Leon, Marta
Kim, Cheol Sang	Kruth, Jean-Pierre	Leong, K.C.
Kim, Do Kyung	Kučerka, Norbert	Lepkova, Katerina
Kim, Dong-Joo (Daniel)	Kukhtarev, Nickolai	Leung, Wallace Woon-Fong
Kim, Dong-Won	Kumar, Deepak	Levänen, Erkki
Kim, Hak Yong	Kusema, Bright	Lewis, Gladius
Kim, Hee Taik	Kuwahara, Yasutaka	Li, Guoqiang
Kim, Jaehwan	Kwong, Philip C.W.	Li, K.Y.
Kim, Kwang S.	Lacki, Piotr	Li, Xing
KIM, Se-Kwon	Ladanov, Mikhail	Lianos, Panagiotis
Kim, Woong	Ladisch, Michael	Lin, I-nan
Kim, Yoong Ahm	Lago, Ana Belén	Lin, Jing-Jenn
Kinoshita, Hajime	Lagoudas, Dimitris	Lin, Shawn D.
Kinuthia, J.M.	Lai, Cheng-Lee	Lin, Tong
Kitagawa, Hiroshi	Lai, Yi Sheng	Lin, Wei
Klenk, Reiner	Laity, Peter	Lind, Anna
Klepka, Andrzej	Lalevée, Jacques	List, Emil J.W.
Klusemann, Benjamin	Lamastra, F.R.	Liu, Huinan
Knezevic, Marko	Lancelot, Christine	Liu, Juewen
Ko, Seung Hwan	Lanceros-Mendez, Senen	Liu, Xinyu
Koch, Reinhold	Landskron, Kai	Lloyd, Natalie
Kofinas, Peter	Langer, Jerzy J.	Lo, Chien-fong
Koissin, Vitaly	Lara-Sanchez, Agustin	Löbmann, Peer
Kojima, Yoshiyuki	Lasia, Andrzej	Lokuge, Weena
Kolpak, Alexie	Lavorgna, Marino	Lomov, Stepan
Komnitsas, Kostas	Law, David	Lopes-Da-Silva, J.A.
Komsa, Hannu-Pekka	Lebaron, Richard	López De Lacalle Marcaide,
Kondoh, Katsuyoshi	Lebedev, Yuri	Luis Norberto
Konegger, Thomas	Leclerc, Corey	López-Álvarez, Miriam
Koos, A.A.	Lee, Chi H.	López, Enrique Vera
Koschny, Thomas	Lee, Jaeyoung	Lorenzo, E.
Kotadia, Hiren	Lee, Jiunn-fwu	Lu, Li
Kotnarowska, Danuta	Lee, Jung-Kun	Lu, Xianmao
Koubaa, Ahmed	Lee, Myoung-Gyu	Luciani, Paola
Kousksou, Tarik	Lee, Rong-Ho	Lugo, M.
Kränkel, Christian	Lee, Woojin	Lugo, Marcos
Kraus, Tobias	Lee, Yong-kyu	Luo, Xiaofan
Kreupl, Franz	Lefebvre, Louis-Philippe	Luque, Rafael
Krishnan, Sadagopan	Leipner, Hartmut	Lusher, Chris
Krokosz, Anita	Lemaître, Aristide	Lvov, Yuri
Krüger, Michael	Lemasson, Fabien	Ma, Anxin
Kruk, Michal	Lenz, Martin	Ma, Z. John
Macquarrie, Duncan	Mayrhofer, Paul	Morales, Gabriel
Madler, L.	Mazur, Ursula	Morandi, Sara
Maffezzoli, Alfonso	Mccarthy, Michael	Moreno-Castilla, Carlos
Magniez, Kevin	McCloy, John	Morent, R.
Mahfuz, Hassan	Mcmurray, Rebecca	Morgiel, Jerzy
Mahmoud, Akrama	Mecerreyes, David	Morigaki, Kenichi
Maierhofer, Christiane	Mehring, Michael	Morin, Jean-françois
Majano, Gerardo	Meinecke, Michael	Morisada, Yoshiaki
Maldonado, Stephen	Meins, Jean-François Le	Morris, Michael
Malekjani, Shokoufeh	Melchionna, Simone	Morrissey, A.J.
Mallada, Reyes	Mely, Yves	Möschwitzer, Jan P.
Malucelli, Giulio	Menaa, Bouzid	Moshkalev, Stanislav
Mandracci, Pietro	Ménard-Moyon, Cécilia	Mottura, Alessandro
Mangwandi, Chirangano	Mendonca, Tania Melo	Moussian, Bernard
Manzoli, Maela	Menendez, J.L.	Moya Corral, Jose Serafín
Marcaccio, Massimo	Menger, Fred	Müller, Bert
Marcal, Helder	Menkara, Hisham	Müller, Carsten
Marder, Michael P.	Merry, Scott	Muller, Robert N.
Marenduzzo, Davide	Meskers, Stefan C.J.	Muñoz-Bonilla, Alexandra
Mariniello, Loredana	Metcalfe, Chris D.	Munoz-Pinto, Dany
Marino, Cyril	Metzler, Ralf	Murthy, S. Narasimha
Markou, I.N.	Meulenberg, Robert W.	Murzin, Dimitry Y.
Marks, Laurence	Michele, Guingand	Musselman, Kevin P.
Marletta, Georgio	Mignardi, Silvano	Musso, Simone
Marotta, Raffaele	Mikael, Skrifvars	Mysliwiec, Jaroslaw
Marr, Patricia C.	Milanese, Chiara	Nadler, Jason
Marschall, Jochen	Millunchick, Joanna Mirecki	Naik, Rajesh R.
Martin, Francisco	Mines, R.A.W.	Nair, Sankar
Martínez, Alberto	Minh, Doan Pham	Najm, Husam
Martins, Rodrigo	Mirzaeifar, Reza	Nakamura, Yoshiaki
Masahashi, Naoya	Mittal, Vikas	Nakashima, Naotoshi
Masami, Ikeyama	Miyachi, Tosiaki	Nakayama, Masaharu
Mascher, Peter	Mizumoto, Tetsuya	Nam, N.D.
Mascia, Leno	Mohamed, Mostafa	Namour, Ph.
Mascolo, Giuseppe	Moini, Mehdi	Nanai, Yasushi
Massart, Thierry J.	Mokaya, Robert	Nara, Yoshitaka
Matos, Maria J.	Molchan, I.S.	Narayan, Roger
Matson, Gerald B.	Montagner, Francisco	Nash, Michael
Matsuda, Yasuhiro H.	Montazami, Reza	Nasrazadani, Seifollah
Matsui, Masaki	Moon, Doo Kyung	Nathal, Michael
Matsukawa, Akihiro	Moortele, Pierre-Francois van de	Nayak, Sashank Sekhar
Matsusaki, Michiya	Morais, S.	Nazarov, Alexey A.
Neher, Dieter	Paipetis, A.S.	Phillion, Andre
Neimark, Alex	Pakharukova, Vera P.	Pichler, Bernhard
Nelson, Toby	Palanisamy, Suresh	Pietrzak, Robert
Neveu, Sophie	Pałys, Barbara	Pillin, Isabelle
Nganga, Sara	Pandrey, Shyam S.	Pinchuk, Anatoliy
Ngo, Ha-duong	Paolone, Annalisa	Pineau, Andre
Nicolas, Rackel San	Papaccio, Gianpaolo	Pingarron, Jose
Nimbalkar, Sanjay	Papini, Marcello	Piorkowska, E.
Nimtz, Günter	Papkovsky, Dmitri B.	Pisano, Aurora Angela
Nishikawa, Masaaki	Papoulis, Dimitrios	Plé, Olivier
Nishiwaki, Tomoya	Parak, Wolfgang J.	Plumeré, Nicolas
Njagi, John	Parikh, Atul N.	Polo, Federico
Nomura, Kotohiro	Park, Chung Hae	Poon, S.J.
North, Michael	Park, Hyung Wook	Popa, Iuliana
Notzel, Richard	Park, Soo-Jin	Pope, Simon A.
Nowack, Bernd	Park, Wanshin	Popescu, Radian
Nugteren, Henk	Parsons, Jason	Potgieter, Herman
Nwauban, Sunny Onyebuchi	Parthasarathy, T.A.	Powrie, William
Obrzut, Jan	Parviz, Babak Amir	Prabakar, K.
Oepen, Hans Peter	Paskevicius, Mark	Prado, Luis
Oezaslan, Mehtap	Patel, Alpesh	Prat, Maria
Ogi, Keiji	Pattersona, Darrell	Prawoto, Yunan
Ogihara, Hitoshi	Paul, Gunther	Principi, Giovanni
Ogris, Manfred	Pavlidis, Ioannis	Prolongo, M.G.
Ohkawa, Kousaku	Pawlak, Andrzej	Provis, John L.
Ohsedo, Yutaka	Payne, Stephen	Pucci, Andrea
Oku, Takeo	Peach, Colin	Puertas, Francisca
Olevsky, Eugene Al	Peel, Matthew	Pujo, Mariacinta C.
Olson, David	Pellegrino, Carlo	Puma, Gianluca
Olsson, Richard	Pellicer, Eva	Qin, Qin
Ombres, Luciano	Pelton, Robert H.	Qin, Wensheng
Onuma, Kazuo	Peng, Zhiwei	Radusch, Hans-Joachim
Ooyama, Yousuke	Penumadu, Dayakar	Radziuk, Darya V.
Opila, Elizabeth J.	Pérez, Marco Antonio	Rae, Cathie
O’Rear, Edgar	Perino, Marco	Ragauskas, Arthur
Oskouei, Reza	Perks, C.M.	Raghunathan, Vijay
Osswald, Sebastian	Persson, Kenneth M.	Rahaman, Saifur
Oudah, Fadi	Petrlova, Jitka	Rahier, Hubert
Özcan, Mutlu	Petukhov, Andrei V.	Rahman, Talat
Pacek, Andrzej	Pezzotti, Giuseppe	Rainer, Rainer
Pacheco-Torgal, Fernando	Phan, Chi M.	Rajan, Ginu
Page, Alister	Phan, Manh-huong	Ramanathan, Rajesh
Ramirez, Anibal	Sagoe-Crentsil, Kwesi	Shi, Xianming
Ramirez, Carmen	Saini, Rajan	Shibata, Takayuki
Ramundo-Orlando, Alfonsina	Sakaushi, Ken	Shieh, Jia-Min
Rana, D.	Sakka, Yoshio	Shim, Jae-hyeok
Rana, Dipak Kumar	Salice, Patrizio	Shin, Hyung Shik
Rangan, B.V.	Sallé, Marc	Shuttleworth, Peter Samuel
Ras, Robin H.A.	Salman, Emre	Sicard, Lorette
Rau, Julietta V.	Sambri, Letizia	Siebenhofer, Matthäus
Rawls, Henry	Sammons, Rachel	Sillanpää, Mika
Reddy, D.V.	Samulski, Edward	Silva, F.J.G.
Reed, Brian E.	Sanada, Yashushi	Silvestroni, Laura
Reeves-Mclaren, Nik	Sanchez, Florence	Singh, Ranjeet
Rego, Ana Maria	Santana, Juan J.	Singh, Thakur Raghu Raj
Rego, Christopher	Sapelkin, Andrei	Sinha, Arijit
Reid, Mike	Sarasini, Fabrizio	Sinner, Eva
Reinhardt, Hans-Wolf	Sarkar, Sohini	Skirtach, André G.
Reiss, Guenter	Sarraf, Hamid	Skovsen, Esben
Ren, J.	Sato, Tsugio	Smet, Philippe F.
Ren, Yuhang	Saulacic, Nikola	Smith, Christopher
Renman, Agnieszka	Sauve, Genevieve	Smith, Alan
Rentsch, C.	Scarpa, Fabrizio	Snoeck, Didier
Riccardi, Leonardo	Schärtl, Wolfgang	Solin, S.A.
Richard, Hans Albert	Scheiner, Stefan	Song, Bo
Rippon, John	Schmidt, Christian W.	Song, Fei
Ritala, Mikko	Schmidt, Daniel F.	Song, Jeong-hoon
Roach, Paul	Schmidt, Michael W.	Song, Myoung Youp
Robben, Lars	Schmitter, Sebastian	Soroushian, Parviz
Robertson, John	Schnepp, Zoe	Sothmann, Björn
Robertson, Will	Scognamiglio, Viviana	Sotiriou, Georgios
Robinson, Sara C.	Sedev, Rossen	Sotiropoulos, S.
Rösch, Notker	Seifalian, A.M.	Spencer, Michelle
Rossi, Fausto	Sen, Tapas	Spiesz, Przemek
Roth, Bradley J.	Sepúlveda-Escribano, Antonio	Spiller, Kara L.
Rouabhia, Mahmoud	Serra, Roger	Srinivasa, Arun
Rowson, Neil	Setlur, Anant A.	Stamatakis, Michail
Ruaan, Ruoh-C.	Setou, Mitsutoshi	Stanek, Chris
Ruckenstein, Eli	Seymour, Kevin	Stang, Peter
Ruffell, Simon	Sha, Wei	Stark, Wolfgang
Russell, Richard	Shah, Vishal Ajit	Steeb, Holger
Sańcheza, Luis	Sheu, Ming-Thau	Stieger, Christof
Sababi, Majid	Shi, Jian	Straumal, Boris B.
Saerens, Lien	Shi, Sheldon	Strawhecker, Kenneth E.
Su, Pi-guey	Telford, Andrew	Vandewal, Koen
Suarez-Garcia, Fabian	Temnov, Vasily	Vantassel, Paul
Suárez, Isaac	Tepavcevic, Sanja	Vanwolleghem, Mathias
Suarez, Sophia N.	Thevuthasan, Theva	Varin, Robert A.
Subbiah, Jegadesan	Thompson, Van P.	Varlamov, Sergey
Subramanian, Vivekanandan	Thomson, Andrew	Vas, Luis
Suh, Yung Doug	Tian, Guiyun	Aashist, Sandeep K.
Sukhishvili, Svetlana A.	Ting, Simon	Vatamanu, Jenel Petrisor
Sulejmani, Sanne	Tjong, S.C.	Vayenas, Dimitris V.
Sun, Andy	Tkachenko, Nikolai	Vecitis, Chad D.
Sun, Danmei	Toal, Vincent	Velasco, J.I.
Sun, Kien Wen	Tong, Minnah	Venezia, A.M.
Sun, Tao	Traisnel, Michel	Venugopal, Jayarama Reddy
Sun, X.S.	Tran, Phuong	Verbeek, Johan
Sung, Shihwu	Travesa, Albert Turon	Verberckmoes, An
Sunol, Joan Josep	Tress, Wolfgang	Verma, Amit
Suryanto, Benny	Trimmel, Gregor	Verron, Elise
Suzuki, Kiyonori	Tringali, Stephane	Viana, Bruno
Swart, Hendrik	Trojan-Piegza, Joanna	Vickery, Karen
Sykora, Richard E.	Trunschke, Annette	Vieille, Benoit
Szczygłowski, Jan	Truss, Rowan	Villa, Carla
Taite, Lakeshia J.	Tsai, Jia-Lin	Vindigni, Vincenzo
Tajber, Lidia	Tsai, Jeff	Viraraghavan, Thiruvenkatachari
Takahashi, Masahide	Tseng, Chun-Chieh	Viscardi, Guido
Takeuchi, Masato	Tseng, W.L.	Visseaux, Marc
Tamburic, Danka	Tsige, Mesfin	Voudouris, Konstantinos
Tanaka, Kunihiko	Tsukada, Keiji	Vozmediano, María
Tanaka, Shunsuke	Tsutsumi, Naoto	Wahrmund, Joshua
Tanaka, Toshiyuki	Tung, Shih-Huang	Wakiya, Naoki
Tang, C.J.	Ubertini, Filippo	Walsh, Darren A.
Tang, Luping	Uda, Nobuhide	Wan, Kai-tak
Taniguchi, Jun	Ulbrecht, Jan S.	Wang, Bin
Tariq, Mohammad	Unnithan, Ranjith R.	Wang, Chengyin
Tasis, Dimitrios	Ushida, Akiomi	Wang, Heming
Tasso, Roberta	Valenzano, Loredana	Wang, Han
Tawil, Bill J.	Vamivakas, Nick	Wang, Jianyun
Tay, Bee Yen	Van Apeldoorn, Aart	Wang, Meng-Jiy
Tayebi, Lobat	Van Den Beucken, Jeroen J.J.P.	Wang, Shaobin
Taylor, Kathryn M.L.	Van Der Voort, Pascal	Wang, Wei-Ning
Teferra, Kirubel	Van Gelderen, P.	Wang, Wen-Xue
Tejchman, Jacek	Van Paassen, Leon	Wang, Xungai
Telfer, Scott	Van Riessen, Arie	Wang, Yaojin
Wang, Zhonglin	Xu, Qingchuan	Zandi, Mohammad
Ward, Liam	Yamaga, Mitsuo	Zarb, George
Warheit, David B.	Yamaguchi, Seiji	Zaumseil, Jana
Warner, Jenifer S.	Yamamoto, Kentaro	Zeng, Hao
Wayne, Steven F.	Yamato, Takehiko	Zhang, Chao
Wee, Andrew Thye Shen	Yamauchi, Satoshi	Zhang, Faming
Wegner, Karsten	Yamauchi, Yusuke	Zhang, Fan
Weidner, Tobias	Yan, Ming	Zhang, Haifei
Wendel, Hans P.	Yang, Eui-Hyeok	Zhang, Haimin
Wennerber, Ann	Yang, H.Y.	Zhang, Mingxing
Wessels, Bruce	Yang, Jenn-Ming	Zhang, Qi
White, Claire	Yang, Ralph T.	Zhang, Qichun
Wilhelm, Manfred	Yang, Rusen	Zhang, XiaoXin
Wilks, Steve	Yang, Yanhui	Zhao, Hengbei
Williams, Gareth	Yazyev, Oleg	Zhao, K.J.
Williams, Jim	Yeh, Jui-Ming	Zharnikov, Michael
Williams, Vance	Yi, Shouliang	Zheng, Minghao
Wong, Danny K.Y.	Yokoyama, Kenji	Zheng, Yuan
Wood, David	Yoshino, Toshihiko	Zhou, Dejian
Worsley, Marcus A.	Young, Steve	Zhou, Jie
Wu, Hwai-Chung	Young, Timothy	Zhou, Qi
Wu, Mingyan	Yousefpour, Ali	Zhou, Zhaoxia
Wu, X.F.	Yousif, Belal F.	Zhu, Junmin
Wünsche, Michael	Yu, Aimin	Zhuo, Chuanwei
Wurmehl, S.	Yu, Hao	Zhuravleva, Ksenia
Xia, Wei	Yu, Xiong	Ziaee, Zainab
Xiao, Boqi	Yuan, Robert	Ziese, Michael
Xiao, Penny	Yun, Hui-suk	Zreiqat, H.
Xie, Zhiwei	Zaghib, K.	Zucchi, Fabrizio
Xin, Hao	Zainuddin, Shaik	Zych, Eugeniusz
Xu, Qiang	Zajickova, Zuzana	

## Notes

*Materials* Editorial Office, MDPI AG, Klybeckstrasse 64, CH-4057 Basel, Switzerland

